# Comparison of oblique lateral lumbar interbody fusion and transforaminal lumbar interbody fusion in the treatment of degenerative lumbar diseases: A protocol for systematic review and meta-analysis

**DOI:** 10.1097/MD.0000000000032356

**Published:** 2022-12-23

**Authors:** Mengqi Na, Xinli Zhan

**Affiliations:** a Department of Spinal Surgery, the First Affiliated Hospital of Guangxi Medical University, Guangxi, China.

**Keywords:** degenerative lumbar diseases, meta-analysis, OLIF, TLIF

## Abstract

Degenerative lumbar diseases are common in the aging population, and is one of the most frequent causes of disability. Lumbar fusion is an effective surgical procedure for alleviating pain and improving function. A variety of fusion techniques applied where necessary are available and the optimal method remains controversial. We performed a protocol for systematic review and meta-analysis to compare the clinical efficacy of oblique lateral interbody fusion (OLIF) and transforaminal lumbar interbody fusion (TLIF) in the treatment of degenerative lumbar diseases.

**Methods:**

This systematic review protocol will be reported in accordance with the Preferred Reporting Items for Systematic Review and Meta-Analyses Protocols (PRISMA-P) 2015 Statement.

Following databases will be searched: PubMed, web of science, MEDLINE, Embase, Cochrane Library, China National Knowledge Infrastructure, Chinese Scientific Journals Database, Wanfang data, and Chinese BioMedicine Literature Database. Only randomized controlled trials comparing OLIF and TLIF for treating degenerative lumbar diseases will be included. The meta-analysis will be performed with Review Manager Version 5.4 software (The Cochrane Collaboration, Copenhagen, Denmark).

**Results:**

The results of this systematic review will be published in a peer-reviewed journal.

**Conclusion:**

This study will elucidate the clinical outcomes of OLIF compared with TLIF in treating degenerative lumbar diseases.

## 1. Introduction

Degenerative lumbar diseases are most prevalent in the elderly and may lead to back pain, radiculopathy, and spinal instability.^[1–3]^ It is among the most common and costly disabling disorders in the United States and continues to increase in prevalence because of the aging population. In the case of clinical deterioration or failed conservative management, surgery should be considered.

Lumbar interbody fusion is a surgical strategy to promote bony arthrodesis by inserting a structural graft into the intervertebral disc space.^[4]^ It can achieve stabilization of the 3-joint complex of the functional spinal unit through pedicle screw fixation.^[5]^ Lumbar interbody fusion is known to have good clinical outcomes, high fusion rates, and low reoperation rates because it has the advantages of a wider fusion bed space and biomechanical anterior column support.^[6]^

As a mini-open anterior retroperitoneal approach, oblique lateral interbody fusion (OLIF) was firstly introduced to treat lumbar degenerative diseases via a physiological corridor between the aorta and psoas in 2012.^[7]^ The mechanism of oblique lateral approach is to achieve indirect neural decompression and lumbar curve correction by placing a larger cage into the disc space.^[8]^ Transforaminal lumbar interbody fusion (TLIF) is considered a standard lumbar fusion technique, providing effective decompression of the neural tissue while avoiding neural injury.^[9]^ Recently, developed techniques of minimally invasive TLIF are associated with minimal tissue trauma, satisfactory clinical improvement, and adequate fusion rate.^[10,11]^ In minimally invasive TLIF, the endoscopic approach helps improve minimalism (i.e., the minimal invasive nature), as suggested by recent reports summarizing clinical experience, clinical case series, and cohort studies.

So far, the effectiveness and safety of OLIF and TLIF in treating degenerative lumbar diseases have not been systematically compared and remain controversial. Therefore, we performed a protocol for systematic review and meta-analysis to compare the clinical and radiographic efficacy of these 2 procedures.

## 2. Methods

### 2.1. Study design

This systematic review protocol will be reported in accordance with the Preferred Reporting Items for Systematic Review and Meta-Analyses Protocols (PRISMA-P) 2015 Statement.^[12]^ The protocol has been registered in PROSPERO (CRD42022371561). Given that the meta-analysis is a secondary research which based on some previously published data, ethical approval is not necessary.

### 2.2. Eligibility criteria

Only randomized controlled trials comparing OLIF and TLIF for treating degenerative lumbar diseases will be included. At least one of the following results is compared: hospital stay, operation time, visual analogue score, Oswestry Disability Index score, incidence of complications and revision rates. The exclusion criteria are as follows: reviews, letters, conferences abstract, case reports or series, comments, and animal experiment.

### 2.3. Electronic searches

A comprehensive search of electronic databases is carried out from the initiation of the databases to November 2022. Following databases will be searched: PubMed, web of science, MEDLINE, Embase, Cochrane Library, China National Knowledge Infrastructure, Chinese Scientific Journals Database, Wanfang data, and Chinese BioMedicine Literature Database. Based on the principle of combination of heading terms and free words, 2 reviewers will manually search of the following terms using Boolean logic (AND, OR, or NOT): “degenerative lumbar diseases,” “oblique lateral lumbar interbody fusion,” “transforaminal lumbar interbody fusion” and “randomized controlled trial.” Disagreements will be resolved through a discussion with a third reviewer. Table 1 presents the retrieval strategy in PubMed.

**Table 1:** Search strategy in PubMed database.

#1 degenerative lumbar diseases [Title/Abstract]
#2 lumbar disc herniation [Title/Abstract]
#3 lumbar spinal stenosis [Title/Abstract]
#4 lumbar spondylolisthesis [Title/Abstract]
#5 lumbar instability [Title/Abstract]
#6 #1 OR #2 OR #3 OR #4 OR #5
#7 oblique lateral lumbar interbody fusion [Title/Abstract]
#8 OLIF [Title/Abstract]
#9 #7 OR #8
#10 transforaminal lumbar interbody fusion [Title/Abstract]
#11 TLIF [Title/Abstract]
#12 #10 OR #11
#13 randomized controlled trial [Publication Type]
#14 randomly [Title/Abstract]
#15 randomized [Title/Abstract]
#16 #13 OR #14 OR #15
#17 #6 AND #9 AND #12 AND #16

OLIF = oblique lateral interbody fusion, TLIF = transforaminal lumbar interbody fusion

### 2.4. Selection of studies

**Figure 1.**
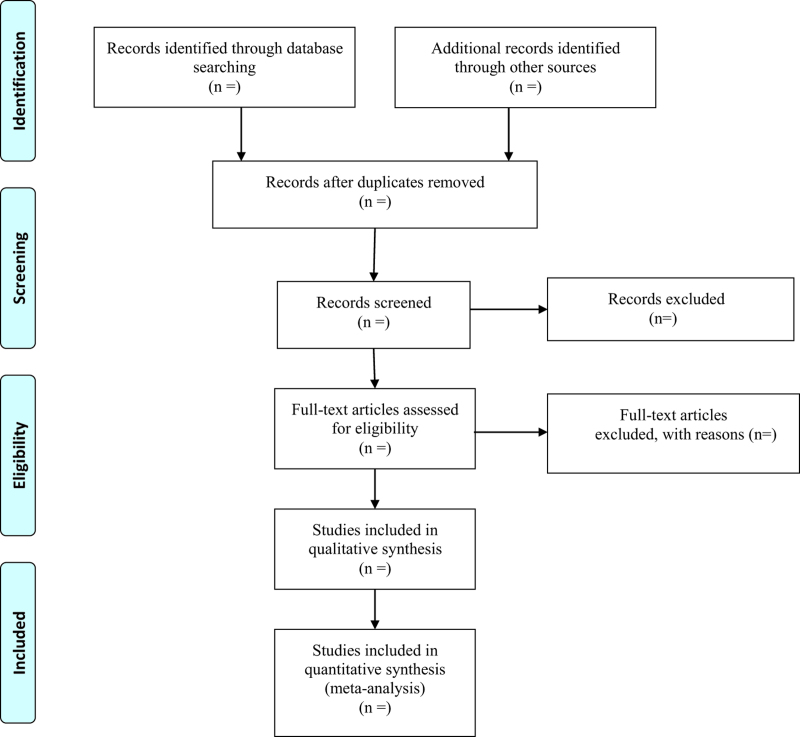
Flow diagram of study selection.

All retrieved literature that meets the requirements will be imported into Endnote X9 software (Camelot UK Bidco Limited, London, United Kingdom). Firstly, 2 researchers will independently screen the titles and abstracts to identify the potentially eligible articles. Secondly, disqualified literature will be removed based on the inclusion criteria through downloading and reviewing the full text. The reasons for exclusion should be recorded at the same time. Finally, the final eligible articles selected will be carefully cross-checked by 2 researchers. Any disagreements will be mediated via a third investigator to reach a consensus. The process of studies selection according to the PRISMA flow chart is illustrated in Figure 1.

### 2.5. Data extraction

Two researchers will independently extract data according to a standard data extraction form by using Excel2013 software. The details of the form are as follows: basic information of studies (authors, title, and publication year), participants characteristics (age, gender, numbers, and course of disease), interventions, outcome indicators, adverse events, etc. If there is any incomplete information in the study, we will contact the corresponding author. All disagreements will be resolved by consulting a third researcher.

### 2.6. Quality assessment

#### 2.6..1. Risk of bias assessments

Two independent investigators will assess the risk of bias, and each trial will be scored as high, low, or unclear risk based on the Cochrane Collaboration Risk of Bias Tool.^[13]^ Two independent investigators evaluate methodological quality of data covers the following 7 domains: random sequence generation (section bias), allocation concealment (section bias), blinding of participants and personnel (performance bias), blinding of outcome assessment (detection bias), incomplete outcome data (attrition bias), selective reporting (reporting bias), other biases.

#### 2.6..2. Quality of evidence assessment

Two independent investigators assessed the level of the quality of evidence with primary outcomes based on grading of recommendations assessment, development, and evaluation system for rating the quality of evidence and strength of recommendations.^[14]^

### 2.7. Data synthesis

The meta-analysis will be performed with Review Manager Version 5.4 software (The Cochrane Collaboration, Copenhagen, Denmark). The mean difference or standardized mean differences will be to adopted measure the effect for continuous variables. Risk ratios or odds ratio will be assumed to calculate the curative effect for dichotomous variables, with both having 95% confidence intervals. *I*^2^ test is be used to determine the heterogeneity. The study is considered to be homogeneous when *I*^2^ ≤ 50%, *P* ≥ .1, a fixed effect model is selected for meta-analysis. Otherwise, if *I*^2^ > 50%, *P* < .1, there is significant statistical heterogeneity in the study and the random-effect model will be used.

## 3. Discussion

Most adults will encounter low back pain amid their lifetime, with the greater part of these situations settling or improving without sequelae.^[15,16]^ For the modest number of patients with severe, persistent pain, lumbar fusion might be required, especially when attendant leg pain or deformity is existing. A variety of fusion techniques applied where necessary are available.^[17,18]^ The efficacy of OLIF and TLIF for the management of degenerative lumbar diseases has been demonstrated in several studies.^[19,20]^ However, based on the published literature, it is difficult to draw a conclusion regarding whether OLIF is superior to TLIF because of small sample sizes, heterogeneity of study objectives and evaluation indices, and a low level of evidence. This study will provide reliable evidence related to the issues. Further multicenter, large sample prospective randomized trials with long-term follow-up periods are warranted for a more comprehensive evaluation.

## Author contributions

**Data curation:** Xinli Zhan.

**Writing – original draft:** Mengqi Na.

**Writing – review & editing:** Xinli Zhan
